# Evaluation of Retinal and Choroidal Thickness in Fuchs' Uveitis Syndrome

**DOI:** 10.1155/2016/1657078

**Published:** 2016-08-08

**Authors:** Ozlem Balci, Mustafa Ozsutcu

**Affiliations:** Ophthalmology Department, School of Medicine, Istanbul Medipol University, 34214 Istanbul, Turkey

## Abstract

*Purpose.* We aimed to investigate retinal and choroidal thickness in the eyes of patients with Fuchs' uveitis syndrome (FUS).* Methods.* Fifteen patients with unilateral FUS and 20 healthy control subjects were enrolled. Spectral domain optical coherence tomography (Spectralis HRA+OCT, 870 nm; Heidelberg Engineering, Heidelberg, Germany) was used to obtain retinal and choroidal thickness measurements. The retinal nerve fiber layer (RNFL) thickness, macular thickness, and choroidal thickness of the eyes with FUS were compared with the unaffected eye and the eyes of healthy control subjects.* Results.* The mean choroidal thickness at fovea and at each point within the horizontal nasal and temporal quadrants at 500 *μ*m intervals to a distance of 1500 *µ*m from the foveal center was significantly thinner in the affected eye of FUS patients compared with the unaffected eye of FUS patients or the eyes of healthy control subjects. However, there were no significant differences in RNFL or macular thickness between groups.* Conclusions.* Affected eyes in patients with FUS tend to have thinner choroids as compared to eyes of unaffected fellow eyes and healthy individuals, which might be a result of the chronic inflammation associated with the disease.

## 1. Introduction

Fuchs' uveitis syndrome (FUS) is an intraocular inflammatory condition that is unilateral in about 90% of cases and involves the vitreous humor, lens, optic disc, and anterior segment [1]. FUS accounts for 2–11% of cases of uveitis, and 2–17% of patients with anterior uveitis have FUS [2–5]. Although the trigger of the inflammation remains elusive, many genetic, sympathetic, immunological, and vascular theories, as well as associations with toxoplasma and toxocariasis, have been proposed [6–14]. Recent studies also show evidence of a viral etiology such as herpes simplex virus or rubella virus in some cases of FUS [15–20]. Diagnostic criteria include diffusely scattered stellate granulomatous keratic precipitates, chronic low-grade anterior chamber reactions, iris stromal atrophy with or without heterochromia, vitreous cells and debris, absence of posterior synechiae, and cystoid macular edema [21]. Pathological studies show a combination of inflammatory, degenerative, and atrophic changes. The iris and ciliary body show low-grade chronic inflammatory cell infiltration of lymphocytes and plasma cells. Although lymphocytes are the predominant infiltrating cells, plasma cells, eosinophils, mast cells, and Russell bodies have all been described. The iris and ciliary body are atrophic with fibrosis and obliteration of the vascular endothelium and a reduced number of melanocytes. Furthermore, degenerative changes are observed in the inner wall of Schlemm's canal and in nerve fibers [22, 23].

Although FUS is classified as an anterior uveitis, studies showed involvement of the posterior segment [24–27], including optic disc hyperfluorescence, peripheral vascular leakage, vitreoretinal modifications such as hyperreflective dots in the vitreous humor and on the retinal surface, thickening of the posterior hyaloid, posterior vitreous detachment, vitreoretinal traction, and epiretinal membrane. Changes in the posterior segment can be observed by indocyanine green angiography (ICGA) and fluorescein angiography. Although ICGA is useful for visualizing the choroidal vasculature, it is invasive and difficult to perform repeatedly. Furthermore, it does not allow sufficient cross-sectional imaging of the choroid. Therefore, optical coherence tomography (OCT) and enhanced depth imaging- (EDI-) OCT are alternative, noninvasive methods of investigating the retina and choroidal space, the latter with the capability of imaging deeper choroidal structures, enabling quasi-quantitative measurement of its thickness [28]. To our knowledge, only one study has evaluated retinal and choroidal parameters in FUS with spectral domain- (SD-) OCT [29].

Therefore, in the present study, we used SD-OCT to evaluate retinal and choroidal thickness in the affected eye of FUS patients compared with the unaffected eye and the eyes of age-, sex-, and refractive equivalent-matched healthy control subjects.

## 2. Materials and Methods

### 2.1. Study Design and Participants

We reviewed the medical records of 15 patients with unilateral FUS and 20 healthy control subjects who were seen at the Ophthalmology Department, School of Medicine, Istanbul Medipol University. This study was approved by the local ethics committee (approval number: 106-2016) and adhered to the tenets of the Declaration of Helsinki. Detailed written informed consent was obtained from all subjects.

Data including age, gender, ocular and medical history, ophthalmic examination, laboratory work-up, and OCT parameters were retrieved from a computerized patient data. Complete ophthalmic examinations were performed including best-corrected visual acuity (BCVA) on Snellen chart, slit-lamp biomicroscopic examination, intraocular pressure measurement by Goldmann applanation tonometer, and fundoscopy with dilated pupils. Diagnosis of FUS was based on the criteria of Kimura et al. [30], including the presence of small, white, diffuse stellate keratic precipitates (KP) on the corneal endothelium; mild anterior chamber cells and flare; lack of iridocapsular posterior synechiae; vitreous disorders such as floaters, vitreous debris, and vitreous cells (63–88% of cases); glaucoma (9–59% of cases); and iris atrophy with or without heterochromia. When the presentation was not typical of FUS, we conducted laboratory tests including complete blood count, sedimentation rate analysis, angiotensin-converting enzyme and serum lysozyme levels, purified derivative skin tests, venereal disease tests, and imaging, such as thoracic computed tomography and magnetic resonance imaging, to exclude other causes of anterior or intermediate uveitis. The ora serrata and peripheral retina were examined to rule out snow banking in eyes with significant vitreous inflammation. Exclusion of other anterior uveitis entities such as herpetic uveitis and Posner-Schlossman syndrome was carried out with the absence of a history of recurrent unilateral inflammatory attacks, especially with an acute elevation of the IOP during inflammatory episodes, absence of patchy or sectoral iris atrophy, distorted pupil, or spiraling of the iris. Typically microgranulomatous KPs with diffuse spread-out disposition on whole endothelium and presence of vitritis were diagnostic for FUS.

Exclusion criteria were as follows: eyes with a refractive error greater than ±3 diopters (D), eyes with glaucoma or ocular hypertension, eyes with dense cataract or media opacity obscuring the accurate visualization of the posterior segment, history of ocular surgery, presence of a coexisting ocular or systemic disease, and use of any topical or systemic medications. The control group consisted of age-, sex-, and spherical equivalent-matched healthy control subjects who visited our outpatient ophthalmology clinic for routine ophthalmic examination.

### 2.2. Examination Protocol and Study Measurements

**Figure 1:**
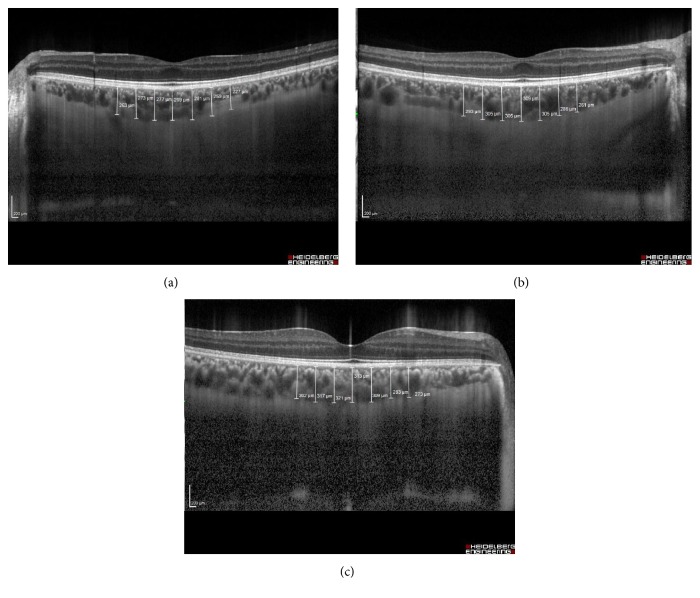
Representative EDI-OCT images of the choroid of a patient with FUS and a healthy control subject. (a) Choroidal thickness of the affected eye in a patient with FUS. (b) Choroidal thickness of the unaffected eye of the same patient with FUS. (c) Choroidal thickness of the eye in a healthy control subject.

The thicknesses of the retinal nerve fiber layer (RNFL), macula, and choroid were measured by OCT (Spectralis HRA+OCT, 870 nm; Heidelberg Engineering, Heidelberg, Germany). Scans for all participants were performed with pupillary dilatation under the same intensity as dim room lighting and were performed by the same experienced technician. All OCT scans were performed at the same time of the day, in the morning, to avoid diurnal fluctuations. An internal fixation target was also used in all scans with a real-time eye tracking system to adjust for eye motion. The RNFL thickness was measured around the disc consecutive circular B-scans (diameter of 3.5 mm). The RNFL thickness (from the inner margin of the internal limiting membrane to the outer margin of the RNFL layer) was automatically segmented using Spectralis software version 6.3.2.0. Average RNFL was used for analysis. Macular thickness was reported in a modified Early Treatment of Diabetic Retinopathy Study macular map with the central foveal subfield which is 1 mm in diameter and the inner and outer subfields having diameters of 3 mm and 6 mm, respectively. EDI-OCT imaging was performed using a method described previously [28]. A 30-degree horizontal section was obtained, going directly through the foveal center and encompassing the macula. Choroidal thickness (CT) was defined as the vertical distance from the hyperreflective line of Bruch's membrane to the hyperreflective line of the inner surface of the sclera. The scan was measured at the fovea and within the horizontal nasal and temporal quadrants at 500 *μ*m intervals to a distance of 1500 *µ*m from the foveal center (Figure 1). CT measurements were performed by the same ophthalmologists with the manual caliper tool of the OCT software and the average of the two measurements was taken for analysis. All images captured had a signal quality of at least 20 dB.

### 2.3. Statistical Analysis

Averaging the measurements of RNFL thickness, macular thickness, and choroidal thickness was used for the analysis. Statistical analysis was performed with SPSS for Windows 17.0 (SPSS Inc., Chicago, IL). Data was recorded as mean ± standard deviation (SD). Normality of data was confirmed using Kolmogorov-Smirnov test. Quantitative data was analyzed using ANOVA and post hoc Bonferroni test for comparison of the means of the three groups. An independent *t*-test and Chi-square test were used to compare variables between patients with FUS and healthy control subjects. RNFL thickness, macular thickness, and choroidal thickness were compared between the affected eyes of FUS patients, the unaffected eyes of FUS patients, and the eyes of healthy control subjects. For control subjects, right eye was selected for the analysis. A value of *p* < 0.05 was considered statistically significant.

## 3. Results

A total of 15 patients with FUS (eight females and seven males) and 20 healthy control subjects (10 females and 10 males) were included in this study. The demographic analysis of groups is presented in Table 1. The mean age in patients with FUS was 36.2 ± 8 years (range, 25 to 42) and it was 35.5 ± 6.2 years (range, 25 to 42) in healthy control subjects, which was statistically insignificant (*p* = 0.41). Gender differences in both groups were statistically insignificant (*p* = 0.56). Mean refractive error in the uveitic eye and in the fellow eye was 1.62 ± 1.2 D and 1.65 ± 0.8 D, respectively. Mean refractive error in control subjects was 1.70 ± 1.1 D. There was no statistically significant difference between patients with FUS and healthy control subjects in terms of refractive error (*p* = 0.61).

Clinical diagnosis of FUS was made at the initial visit. The most frequent presenting symptom was ocular discomfort, which was reported by seven patients. Five patients were aware of the presence of heterochromia. Three patients complained about blurred vision. Ocular findings are shown in Table 2.

Iris stromal atrophy was present in five patients. Heterochromia was present in seven patients. Loss of iris crypts was noted in all patients. Posterior subcapsular cataract was present in three patients. Small- to-medium sized stellate keratic precipitates and anterior chamber reaction were noted in all patients. Iris nodules, including Koeppe and Busacca nodules, were observed in five patients. No retinal lesions or scars from prior toxoplasmosis were seen in any patient, although varying degrees of vitreous cells or debris were observed in all patients. Best-corrected visual acuity (BCVA) was ≥0.8 in 12 patients and <0.7 in three patients. The cause of diminished BCVA was cataracts in all cases.

Retinal and choroidal thicknesses are presented in Table 3.

### 3.1. RNFL Thickness

Average RNFL thickness was 108 ± 12.1 *μ*m in the affected eyes of FUS patients, 109 ± 14.9 *μ*m in the unaffected eyes of FUS patients, and 110 ± 14.2 *μ*m in the eyes of healthy control subjects (Table 3). No significant differences in RNFL thickness were observed between the affected and unaffected eyes of FUS patients (*p* = 0.12) or between the affected eyes of FUS patients and the eyes of control subjects (*p* = 0.15).

### 3.2. Macular Thickness

Central foveal thickness was 251.7 ± 29.2 *μ*m in the affected eyes of FUS patients, 254.5 ± 23.1 *μ*m in the unaffected eyes of FUS patients, and 255.1 ± 21.2 *μ*m in the eyes of healthy control subjects (Table 3). There were no significant differences in central foveal thickness between the affected and unaffected eyes of FUS patients (*p* = 0.17) or between the affected eyes of FUS patients and the eyes of control subjects (*p* = 0.11). Likewise, there were no significant differences in inner and outer macular thicknesses between groups (*p* > 0.05 for all comparisons).

### 3.3. Choroidal Thickness

Representative EDI-OCT images of the choroid of a patient with FUS and a healthy control subject are shown in Figure 1. Choroidal thickness at fovea and at each point within the horizontal nasal and temporal quadrants was significantly thinner in the affected eyes of FUS patients compared to the unaffected eyes (*p* < 0.05 for each comparison). Similarly, choroidal thickness at fovea and at each point within the horizontal nasal and temporal quadrants was significantly thinner in the affected eyes of FUS patients compared to the eyes of healthy control subjects (*p* < 0.05 for each comparisons). However, choroidal thickness at fovea and at each point within the horizontal nasal and temporal quadrants was also similar between the unaffected eyes of FUS patients and the eyes of healthy control subjects (*p* > 0.05 for each comparison).

## 4. Discussion

In the present study, we compared retinal nerve fiber layer and macular and choroidal thickness between the affected eyes of FUS patients, the unaffected eyes of FUS patients, and the eyes of age-, sex-, and spherical equivalent-matched healthy control subjects. We found choroidal thinning at fovea and at each point within the horizontal nasal and temporal quadrants in the affected eyes of FUS patients compared with the unaffected eyes of FUS patients or the eyes of control subjects, whereas there was no statistically significant difference in RNFL and macular thickness values.

Although FUS was first described in 1906, its etiology remains unknown. In FUS patients, chronic low-grade anterior segment inflammation can persist for years, leading to various degrees of atrophy of the iris and ciliary body. The vascular layer of the eye, the choroid, contains choroidal vessels, connective tissue, and melanin. Large-diameter vessels are located in the outermost layer of the choroid, and medium-sized vessels lie between the large-diameter vessels and choriocapillaris. The choroid is more vulnerable to the effects of the inflammatory and vascular systemic diseases than are other tissues. As the choroid plays an important role in the pathogenesis of many diseases of the posterior segment of the eye, imaging choroidal structure is important for understanding the pathophysiology of these diseases. Although ICGA, laser Doppler flowmetry, and B-mode ultrasonography have been used for many years to detect choroidal vessel defects and circulation changes, choroidal thickness, and gross choroidal abnormalities, none of these techniques provide cross-sectional images of the anatomy of the retinal pigment epithelium or choroidal layers to allow accurate assessment of choroidal thickness and morphology. Described by Spaide and collaborators [28], EDI-OCT is an imaging technique using SD-OCT devices which enables cross-sectional, high-resolution visualization of the choroid in a simple, reproducible, and noninvasive manner and provides a better understanding of choroidal changes that occur in many diseases.

Choroidea is influenced during the inflammatory processes, especially in posterior uveitis. Many studies have investigated choroidal abnormalities resulting from various acute and chronic ocular inflammatory conditions [31–39]. These studies demonstrated that acute and chronic inflammation can show different effects on choroidea. Nakayama et al. showed that choroidal thickness, as measured by EDI-OCT, can serve as a marker of the degree of choroidal inflammation in acute Vogt-Koyanagi-Harada disease [31]. Similarly, Maruka et al. demonstrated that the choroid is thicker during the acute stage of Vogt-Koyanagi-Harada disease [32]. Ishikawa et al. found an increase in subfoveal choroidal thickness during the acute phase of uveitis in patients with Behçet's disease and showed that choroidal thickness correlates with anterior and posterior ocular inflammation scores [33]. Kim et al. also found an increase in subfoveal choroidal thickness in the acute phase of Behçet's posterior uveitis [34]. Multiple studies suggest that increased blood flow due to acute inflammation and choroidal effusion is the mechanism responsible for choroidal thickening in ocular inflammation [35, 36]. However, Coskun et al. reported thinning of subfoveal choroidal tissue in patients with Behçet's uveitis, perhaps because chronic inflammation and resulting ischemic changes could induce fibrosis [37]. Similarly, Maneschg et al. found significant thinning of the choroid after endophthalmitis-induced chronic inflammation that was associated with decreased choroidal perfusion [38].

Choroidal thickness varies depending on its location relative to the macula; it is thinnest in the nasal area, thickest in the subfoveal area, and thin in the temporal area. Two previous studies reported mean subfoveal choroidal thickness of 287 *μ*m and 332 *μ*m in normal eyes [28, 39]. In the present study, mean subfoveal choroidal thickness in the unaffected eyes of FUS patients and the eyes of healthy control subjects was 313.6 ± 26.8 *μ*m and 318 ± 40.1 *μ*m, respectively. Therefore, our findings are consistent with those of previous studies and indicate that the values of subfoveal choroidal thickness in the present study are within the normal ranges.

To the best of our knowledge, only one study has assessed retinal and choroidal thickness using SD-OCT in FUS patients. A recent retrospective study by Kardes et al. demonstrated that mean ganglion cell complex thickness and subfoveal choroidal thickness in the affected eyes of patients with FUS are reduced compared with the unaffected eyes, whereas the RNFL thickness and macular thickness were not different between eyes. In the present study, we also found thinner choroidal thickness at fovea and at each point within the horizontal nasal and temporal quadrants at 500 *μ*m intervals to a distance of 1500 *µ*m from the foveal center in the affected eyes compared with the uninvolved fellow eyes and healthy eyes of the control subjects. We speculate that chronic inflammation may affect choroidal perfusion or induce choroidal fibrosis and thereby reduce choroidal thickness in FUS. Some limitations of the present study must be also considered. Limitations of our study included the relatively small number of patients participating in the study. Additionally, we have only evaluated the choroid by EDI-OCT. The most recent advance in OCT technology, known as swept-source OCT (SS-OCT), further improves upon the precision with which we can determine the inner and outer boundaries of the choroid, while also allowing examination of the choriocapillaris and larger choroidal vessels. SS-OCT permits a wider range of imaging. Moreover, we did not perform ICGA in the present study. ICGA and longitudinal studies would help advance our understanding of the effect of chronic inflammation on the choroid in FUS.

In conclusion, affected eyes in patients with FUS tend to have thinner choroids as compared to eyes of unaffected fellow eyes and healthy individuals, which might be a result of the chronic inflammation associated with the disease. Further studies with large sample sizes and advanced imaging technology would be required to determine our observations in the structural changes of choroidea in FUS.

## Figures and Tables

**Table 1:** Demographic data of patients and healthy subjects.

Demographics	Patients with FUS	Healthy subjects
Male	8	10
Female	7	10
Mean age (years ± SD)	36.2 ± 8	35.5 ± 6.2
Mean refractive error (SE)	+1.63 ± 1.2	+1.70 ± 1.1

SD: standard deviation; SE: spherical equivalent.

**Table 2:** Characteristics of patients with Fuchs' uveitis syndrome.

Clinical signs	Number of eyes	% eyes
Laterality		
OD	7	46.7%
OS	8	53.3%

Stellate KP	15	100%

Heterochromia	7	46.7%

Iris atrophy	5	33.3%

Iris nodule		
Koeppe	3	20%
Busacca	2	13.3%

Angle vessels	2	13.3%

Russel bodies	2	13.3%

Anterior chamber reaction		
0.5+	5	33.3%
1+	7	46.7%
2+	3	20%

Cataract	3	20%

Vitreous cells and debris	15	100%

**Table 3:** Retinal and choroidal thicknesses in patients with FUS and healthy control subjects.

OCT parameters	Eyes with FUS	Uninvolved eyes	Healthy eyes
*RNFL thickness (µm)*			
(i) *Average RNFL *	108 ± 12.1	109 ± 14.9	110 ± 14.2

*Macular thickness (µm)*			
(i) Central foveal thickness	251.7 ± 29.2	254.5 ± 23.1	255.1 ± 21.2
(ii) Inner macular thickness	313.8 ± 27.2	315.8 ± 26.2	316.8 ± 30.1
(iii) Outer macular thickness	286.6 ± 25.6	287.6 ± 23.8	290.3 ± 25.6

*Choroidal thickness (µm)*			
(i)* Foveal center (µm)*	276.7 ± 22.9	313.6 ± 26.8	318 ± 40.1
(ii) *Nasal (500 µm)*	274.3 ± 26.8	310.5 ± 25.9	315 ± 42.9
(iii) *Temporal (500 µm)*	276.8 ± 27.9	305.8 ± 19.9	307 ± 32.5
(iv) *Nasal (1000 µm)*	265.2 ± 27.6	302.3 ± 28.1	305.1 ± 35.9
(v) *Temporal (1000 µm)*	265.6 ± 28.9	300.7 ± 27.5	300.2 ± 35.1
(vi) *Nasal (1500 µm)*	255.5 ± 37.9	295.3 ± 26.2	298.1 ± 52.9
(vii) *Temporal (1500 µm)*	245.4 ± 46.5	285.1 ± 25.9	289.3 ± 22.5
